# CSF Rhinorrhea: A Rare Clinical Presentation of Choroid Plexus Papilloma

**DOI:** 10.3390/curroncol28010073

**Published:** 2021-01-31

**Authors:** Layth Mula-Hussain, Julia Malone, Marlise P. dos Santos, John Sinclair, Shawn Malone

**Affiliations:** 1 Radiation Oncology Division, The Ottawa Hospital—University of Ottawa, Ottawa, ON K1H 8L6, Canada; julmalone@ohri.ca (J.M.); smalone@toh.ca (S.M.); 2 Department of Medical Imaging, The Ottawa Hospital—University of Ottawa, and Ottawa Hospital Research Institute, Ottawa, ON K1H 8L6, Canada; msantos@toh.ca; 3 Neuro-Surgery Division, The Ottawa Hospital—University of Ottawa, Ottawa, ON K1Y 4E9, Canada; jsinclair@toh.ca

**Keywords:** choroid plexus tumour, cerebellopontine angle, CSF rhinorrhea, stereotactic radiosurgery

## Abstract

Choroid plexus papilloma (CPP) is a rare brain tumour occurring mostly in infants and children. Most CPPs are intraventricular and present with symptoms and signs of increased intracranial pressure (ICP). This case report describes a middle-aged female who presented with spontaneous cerebrospinal fluid (CSF) rhinorrhea from a tumour located in the cerebellopontine angle (CPA). She underwent craniotomy with subtotal tumour resection and remained progression and rhinorrhea-free for several years. Upon clinical progression, the patient was treated with Cyberknife stereotactic radiosurgery. The patient clinically improved and demonstrated a favourable radiologic response to radiosurgery.

## 1. Introduction

Choroid plexus tumour (CPT) is a rare epithelial intracranial extra-axial brain tumour that usually grows inside ventricles and is connected to the choroid plexus or near the natural openings of the ventricles. The annual incidence is 0.3 cases per million [1], and they account for about 0.5% of all intracranial neoplasms. CPT can be classified as choroid plexus papilloma (CPP, WHO grade I) or choroid plexus carcinoma (CPC, WHO grade III), with a ratio of 5:1, respectively. Approximately 80% of CPCs occur in children [2]. The most common location of CPTs in adults is the fourth ventricle, followed by the cerebellopontine angle (CPA) [3,4]. Clinically, CPT tends to cause hydrocephalus, and patients present with symptoms of raised intracranial pressure. The treatment of choice is gross total resection (GTR) [5], when clinically feasible. Neurosurgeons must assess the pros and cons of GTR versus the potential morbidity of complete excision. Historically, radiotherapy has been used in cases where surgery is not possible or ended up with subtotal resection, after local recurrence, or in high-grade CPC [6]. Adjuvant chemotherapy has been used in CPC; however, its benefit is not clear. The five-year survival rates are reported to be as high as 100% in CPP following complete resection, with low local recurrence rates. CPC grows more rapidly and has a less favourable outcome, with a 5-year survival rate of 26–40% [2]. Improvements in surgical techniques and post-operative care have improved survival [7].

## 2. Case Study

**Figure 1:**
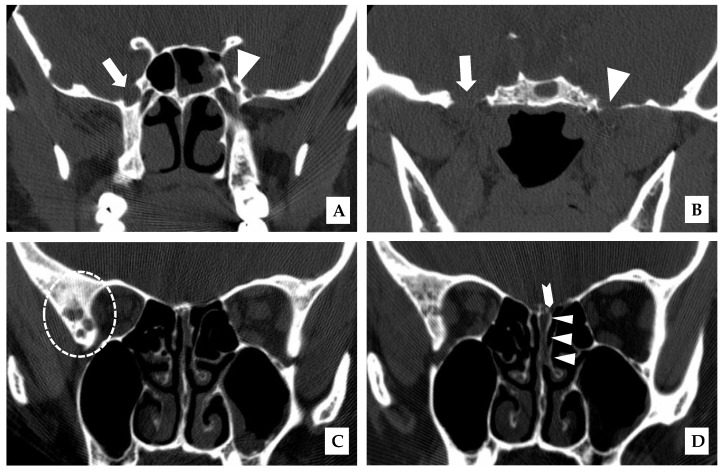
Coronal plane, unenhanced CT scan of the paranasal sinuses obtained with bone window and in prone position. (**A**–**D**, Dec. 2007). (**A**): prominent right (arrow) and normal left (triangle) foramen rotundum; (**B**): prominent right (arrow) and normal left foramen ovale (triangle); (**C**): prominent right sphenoidal arachnoid pits (dashed circle); (**D**): focal defect of the left planum sphenoidale (chevron) and adjacent opacification of the left ethmoid sinus and upper nasal cavity (CSF leak, triangles).

A 43-year-old woman presented in January 2007 with cerebrospinal fluid (CSF) rhinorrhea from the left nostril on bending forward, headache, and later on with ataxia. There was no prior history of skull trauma or surgery and no significant past medical history. After the confirmation of a CSF leak with a β-2 transferrin test, a CT scan of the paranasal sinuses on December 2007 showed the enlargement of the right foramina rotundum and ovale (Figure 1A,B).

**Figure 2:**
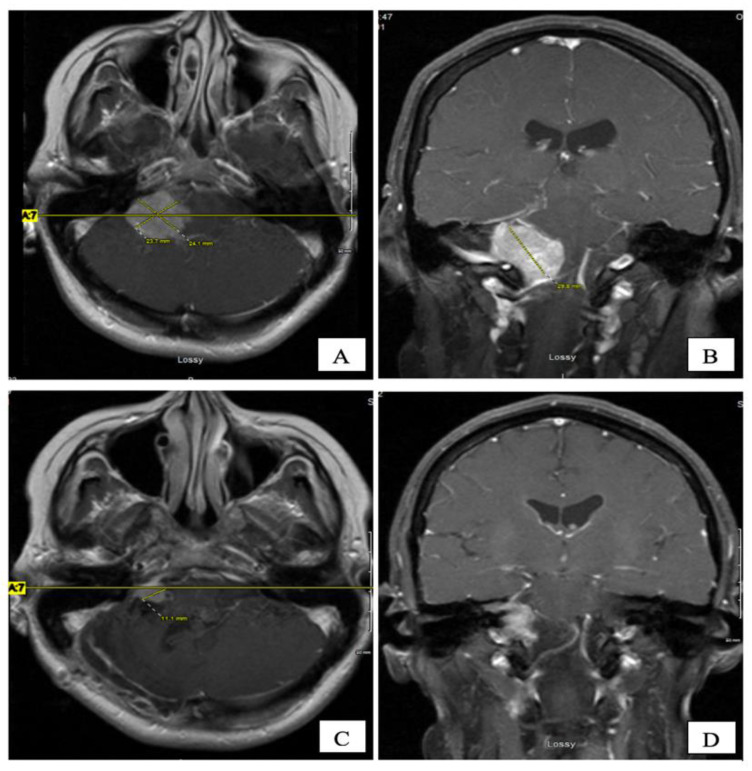
Pre-operative ((**A**) axial, and (**B**) coronal) and 2 months post-operative ((**C**) axial, and (**D**) coronal) post-gadolinium T1-W MRI sequences. The CPA tumour measured 24 × 24 × 30 mm and 11 × 9 mm, respectively.

An MRI of the brain on May 2008 (Figure 2A,B) revealed an enhancing, T1-isointense and T2-hyperintense extra-axial CPA mass lesion measuring 24 × 24 × 30 mm, associated with prominent flow-voids and causing mass-effect on the lower pons and the medulla oblongata. The mass encroached on the cisternal segments of the right 7th and 8th canal nerves complex. Inferiorly, the mass extended up to the level of the foramen magnum. Our retroactive reassessment of the CT scan of the paranasal sinuses identified multiple unilaterally prominent aberrant arachnoid granulations (arachnoid pits) of the right sphenoid bone and the focal disruption of the left planum sphenoidale, contiguous to an abnormal opacification of the medial left ethmoid sinus and upper nasal cavity, in keeping the source of a CSF leak with high intracranial pressure features (Figure 1C,D). The retrospective review of the above mentioned MRI showed a very mild compensated communicating hydrocephalus (Figure 2B).

In September 2008, the patient underwent a right posterior fossa craniotomy with facial nerve monitoring. A frozen section of the tumour revealed a CPP. The tumour was extremely vascular, soft, friable, and adherent to some cranial nerves. A subtotal excision of the tumour was performed in an attempt to preserve cranial nerve function. Final pathology revealed a WHO grade 1 CPP. Histologically, the tumour tissue had a papillary structure with a fibrovascular backbone and was covered by cuboidal to low columnar cells. Postoperatively, her headache and CSF rhinorrhea stopped, but she developed new vocal cord palsy and partial right hearing loss and her ataxia continued. MRI at eight weeks postoperatively revealed encephalomalacia and an 11 × 9 mm residual enhancing tumour in the right internal auditory canal extending into the right CPA without mass effect and resolution of the hydrocephalus (Figure 2C,D). She was kept on annual clinical and radiologic follow-up for over four years, till December 2012, where her annual follow up was discontinued as she was feeling better with stable radiological residual disease.

**Figure 3:**
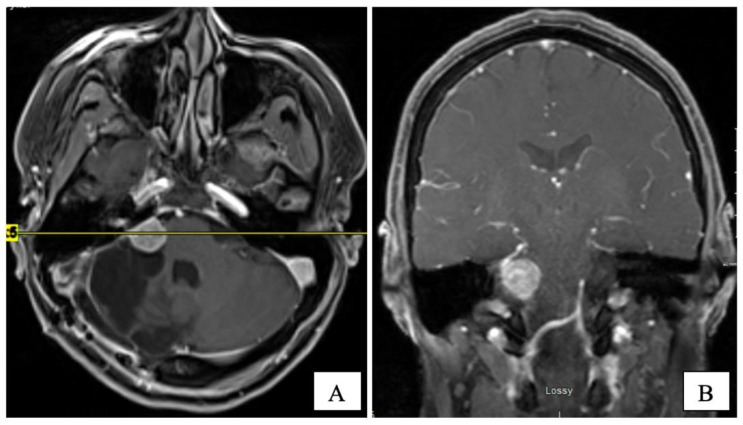
T1-W post-gadolinium MRI at 7 years post-op on axial (**A**) and coronal (**B**) planes. The CPA tumour measured 16 × 15 × 17 mm.

Several years later, in mid-2015, she presented with headaches, worsening ataxia, and new-onset facial palsy. Her physical exam revealed asymmetric pupils (right 5 mm and left 3 mm), reactive to light with a horizontal beat (gaze-evoked), nystagmus, and diplopia on lateral gaze. She had right-side hearing loss and an absent gag reflex. A new MRI revealed the residual tumour’s progression (14 × 14 × 17 mm) with central cystic degeneration/necrosis. The tumour extended further into the right internal auditory canal, causing a mild mass effect on the brainstem. The patient was reluctant to consider further surgical intervention. Three subsequent MRIs performed in 2015 demonstrated the continued slow growth of the residual tumour (Figure 3).

**Figure 4:**
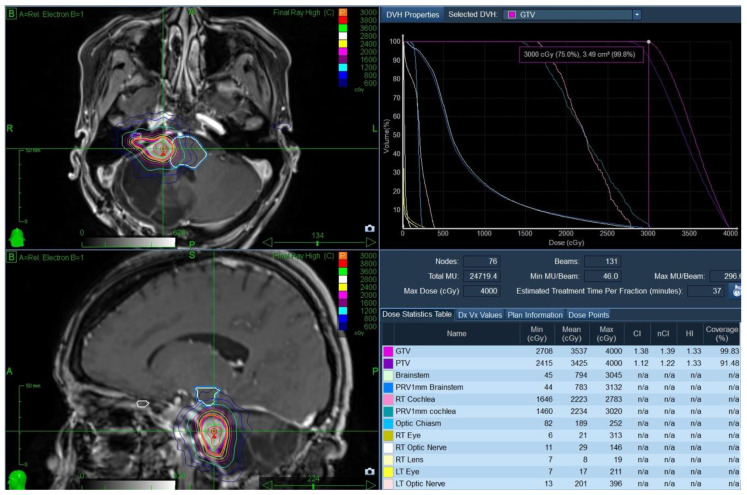
CK SRS treatment plan (January 2016) with the axial and coronal sections, the dose–volume histogram (DVH) and the dose statistics table of the pertinent volumes.

The case was discussed at neuro-oncology rounds. The multidisciplinary consensus was to offer the patient a course of stereotactic radiosurgery (SRS) using the frameless CyberKnife^®^ (CK) as a non-invasive method of tumour control while allowing function preservation. The patient underwent CT/MR simulation and received treatment in January 2016. The gross tumour volume (GTV) was 3.5 cc and was treated to 3000 cGy in 5 fractions (GTV covered by the 99.8% isodose line), with no treatment margin. Treatment was delivered in five daily fractions over one week. A planning target volume was strictly used for treatment planning purposes. The brainstem doses were as follows: Dmax was 3045 cGy, D0.03cc was 2942 cGy, and the D1cc was 2157 cGy (Figure 4). 

**Figure 5:**
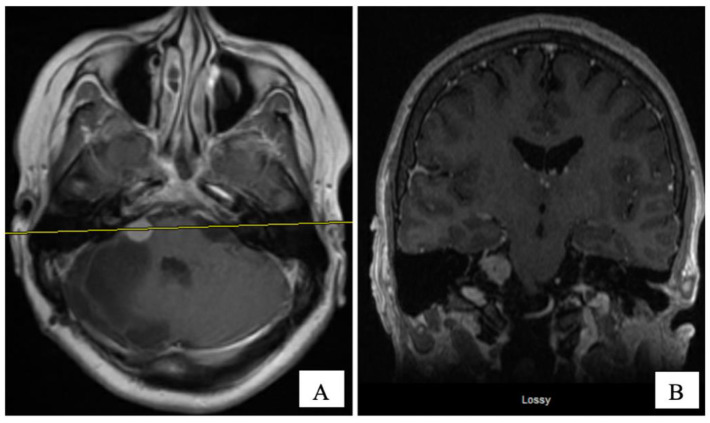
Post-gadolinium T1-W MRI at four years post CK, on axial (**A**) and coronal (**B**) planes. The CPA tumour shrank to 12 × 12 mm.

The patient tolerated SRS well, with no acute or late toxicity. Over the next five years, she had gradual clinical improvement in her gait, swallowing and facial palsy. Additionally, she had complete resolution of her headaches and improvement in her voice function. Serial MRIs revealed her tumour regression with the relief of mass effect on the brainstem (Figure 5). There were no T2/Flair changes (acute or late edema) in the brainstem post-treatment. 

## 3. Discussion

This is a rare case of CPA choroid plexus papilloma presenting with CSF rhinorrhea. Initial imaging revealed a non-specific right CPA tumour and features for long-standing intracranial hypertension. The patient underwent craniotomy and the subtotal excision of her tumour. Pathology revealed a WHO grade I CPP. Seven years after initial surgery, there was a radiologic progression of her tumour, and she developed headaches, worsening ataxia, and new-onset facial palsy. Following a multidisciplinary tumour board, she was treated with salvage CK-based SRS (3000 cGy in 5 fractions). Over the next five years, the patient had gradual subjective clinical improvement. Serial MRIs revealed objective radiological response to SRS. This case highlights two interesting clinical findings: CSF rhinorrhea is a rare presentation from CPP, and CK SRS resulted in the durable local control of this rare tumour.

CPT usually presents with symptoms of raised intracranial pressure (ICP) due to obstructive hydrocephalus or from CSF overproduction [7]. Hosmann et al. reported that adult CPT present with the following symptoms: headache (68%), gait disturbance (53%), vertigo (37%), diplopia (32%), incidental finding (21%), increased ICP (16%), paresis (11%), and behavioural changes (5%) [5]. In our case report, the patient presented with CSF rhinorrhea due to CPP in the CPA region, which to our knowledge, is the first-ever reported presentation in the literature.

CSF leak is caused when there are combined bony and dural defects and a sufficient pressure gradient that allows CSF to drain externally [8]. This can occur at multiple sites, and the predisposing causes include trauma, prior skull base or sinus surgery, chronic increased ICP, and arachnoid granulations [9]. CSF leak has been rarely reported in other CPA tumours, such as schwannomas [10]. Fluid should be collected and sent for β-2 Transferrin, a protein found almost exclusively in CSF, to confirm the presence of a CSF leak. A thin-section multidetector CT scan is used to detect the bony breach’s site and the source of the leak. CT or MR cisternography can also be used to identify the location of a fluid leak. The most common site for spontaneous CSF leak is the cribriform plate, followed by the ethmoid roof/planum sphenoidale. Primary CSF leaks can also involve the sphenoid sinus, peri sella, and pterygoid recesses [11]. In this case report, the CSF leak site was at the ethmoid roof/planum sphenoidale. Due to the constellation of osseous abnormalities found in the CT scan and absence of a history of trauma, we think that the CPP led to over-production of CSF, similar to what Eisenberg et al. reported [12], which caused chronic increased ICP that overtime, remodeled the skull base and caused erosions that led to the CSF leak through the ethmoid roof with CSF rhinorrhea. We think that the unilateral aberrant arachnoid pits/granulations [13], foramen ovale and rotundum result from the chronically increased ICP due to the long-standing CSF overproduction by the CPP.

Gross total or safe-maximal resection is the gold standard of care in the treatment of this benign tumour. At operation, the tumour was compressing the brainstem and cerebellum and extended into the internal porus acusticus. The tumour was adherent to several cranial nerves, including CN VII through CN X. As a result, the patient developed some cranial nerve injuries with clinical deficits, including hearing loss (CN VIII), swallowing dysfunction (CN VII, IX and X), impaired voice function (CNX), impaired gag reflex (CN IX and X) and facial palsy (CN VII). Her voice and swallowing function gradually improved, indicating that these deficits were not permanent.

Wolff et al. reported on 566 patients with CPP [3]. Following complete resection, the 10-year survival rate was 85%, compared to 56% with subtotal excision and a one-year survival rate of 50% in patients after biopsy alone (P = 0.002). In that study, CPT in the CPA region was associated with older age, benign histology, and female gender [3]. Our patient achieved so far more than twelve years from her initial diagnosis (seven years from her subtotal open surgical resection, with an additional five years from her salvage radiosurgery, to date).

The utility of radiation with conventional techniques and dose fractionation is not well documented in the literature [4,14]. SRS has been used to treat CPP for patients with inoperable tumours or following local recurrence. Duke reported the effective use of SRS in pineal CPP with a frame-based Gamma-Knife in 1997 [15]. Over the last two decades, SRS has been an effective strategy to manage resistant CCP, including tumours in the CPA [16]. The optimal SRS dose and fractionation remained to be defined as do rates of long-term tumour control [5]. In-Young Kim et al. reported tumour control in 8 of 11 tumours (73%) after one or more Gamma Knife SRS procedures. In the series, the longest follow-up post-SRS was 41 months [16]. In our case, durable tumour control was achieved over 60 months of follow-up, with no evidence of acute or late SRS toxicity. CK was a safe, well-tolerated, and effective option in recurrent CPP at CPA.

## 4. Conclusions

CSF rhinorrhea is a rare presentation of CPP in the CPA. CPP is usually managed with a definitive maximal-safe surgical resection. Our patient developed clinical and radiologic progression following the subtotal excision of the tumour. Salvage CK resulted in a significant improvement in the patient’s neurologic deficits and durable local tumour control.

## Data Availability

Not available.
